# Using *Trichoderma asperellum* to Antagonize *Lasiodiplodia theobromae* Causing Stem-End Rot Disease on Pomelo (*Citrus maxima*)

**DOI:** 10.3390/jof9100981

**Published:** 2023-09-29

**Authors:** Nguyen Quoc Khuong, Dinh Bich Nhien, Le Thi My Thu, Nguyen Duc Trong, Phan Chan Hiep, Vo Minh Thuan, Le Thanh Quang, Le Vinh Thuc, Do Thi Xuan

**Affiliations:** 1 Faculty of Crop Science, College of Agriculture, Can Tho University, Can Tho 94115, Vietnam; nqkhuong@ctu.edu.vn (N.Q.K.); thule@ctu.edu.vn (L.T.M.T.); nguyenductrongvp4@gmail.com (N.D.T.); pchanhiep28@gmail.com (P.C.H.); thuanvo27022001@gmail.com (V.M.T.); quahgm@gmail.com (L.T.Q.); lvthuc@ctu.edu.vn (L.V.T.); 2 Institute of Food and Biotechnology, Can Tho University, Can Tho 94115, Vietnam; nhien040101@gmail.com

**Keywords:** Lasiodiplodia theobromae, pomelo, Trichoderma asperellum, stem-end rot

## Abstract

Stem-end rot disease has been causing damage to the production of pomelos in Vietnam. The cur-rent study aimed to (i) isolate fungal pathogens causing pomelo stem-end rot disease (PSERD) and (ii) discover *Trichoderma* spp. that had an antagonistic ability against pathogens under in vitro conditions. Fungi causing PSERD were isolated from pomelo fruits with symptoms of stem-end rot disease and collected from pomelo farms in Ben Tre province, Vietnam. Moreover, 50 fungal strains of *Trichoderma* spp. also originated from soils of these pomelo farms in Ben Tre province and were dual-tested with the fungal pathogen on the PDA medium. The results demonstrated that 11 pathogenic fungi causing PSERD were isolated from the fruit and showed mycelial growth of roughly 5.33–8.77 cm diameter at 72 h after inoculation. The two fungi that exhibited the fast-est growth, namely, S-P06 and S-P07, were selected. ITS sequencing of the S-P06 and S-P07 fungi resulted in Lasiodiplodia theobromae. All the 50 *Trichoderma* spp. strains were allowed to antago-nize against the S-P06 and S-P07 strains under in vitro conditions. The greatest antagonistic effi-ciency was found in *Trichoderma* spp. T-SP19 at 85.4–86.2% and T-SP32 at 84.7–85.4%. The two antagonists were identified as *Trichoderma asperellum* T-SP19 and T-SP32. The selected strains of *Trichoderma asperellum* were potent as a biological control for fruit plants.

## 1. Introduction

The genus *Lasiodiplodia* consists of 31 species, most of which have been distinguished molecularly by sequencing conserved genes [1]. Within the *Botryosphaeriaceae* family, this genus is ubiquitous, especially in tropical and subtropical regions of the world [2]. Similarly, most species of the *Botryosphaeriaceae* family are plant pathogens and appear on numerous types of crops, causing fruit rot, stem-end rot, plant withering, and root rot [3]. Species of *Lasiodiplodia* are polymorphological and diverse ascomycetes, and are considered one of the most severe pathogens causing soft rot, and root and fruit rot on perennial fruit plants, spermatophytes, vegetables, and even ornamental plants [4,5]. Moreover, *Lasiodiplodia* fungi are known to cause infections in human beings [6]. Among species of *Lasiodiplodia*, *Lasiodiplodia theobromae* is an economically major pathogenic fungus that causes postharvest decay on many important horticultural fruits. The species *L. theobromae* is known as the cause of dry strawberry fruits in Turkey [7]. In Korea, *L. theobromae* has been reported on mango [8]. Moreover, toxins produced by *L. theobromae* have been associated with infection of mandarin orange (*Citrus nobilis*), lime (*C. aurantifolia*), makrut lime (*C. hystrix*), and winter squash (*C. maxima*) [9]. This is because *L. theobromae* fungi produce substances that soften the leaf surface and bark wall for penetrance but have no role in infection [10]. This postharvest decay can result in remarkable economic losses in the production of some horticultural crops [11]. The optimum growth of *L. theobromae* is at 30 °C and in relatively high humidity [12]. This is close to the weather in Ben Tre province, Vietnam, which features a tropical monsoon climate. Therefore, the disease could significantly affect the production of citrus there, especially pomelos. In addition, numerous chemical fungicides have been reported to be effective against diseases caused by *L. theobromae*, such as thiabendazole (TBZ) and imazalil (IMZ) [13]. However, the exporting standards among markets worldwide are different [14]. Furthermore, improper fungicide usage by farmers raises concerns over its impact on the environment and human health [15]. Therefore, biological approaches are considered promising due to their environmental safety and their antagonistic activities against pathogens [16]. Among the biological approaches, biofungicides formulated from species of *Trichoderma* are widely utilized for controlling diseases in fruit plants. Their direct mechanisms of antagonism are competition, antibiotic production, and parasitism [17]. In fact, *Trichoderma* spp. can effectively control many fungal pathogens, including *Fusarium*, *Rhizoctonia solani*, *Sclerotium rolfsii*, *Botrytis cinerea*, and *Aspergillus niger* [18,19,20,21,22]. Numerous previous studies claimed that the *Trichoderma* genus is efficient in controlling fungal pathogens on crops and directly protects crops from threats [23]. Some studies revealed that *Trichoderma* spp. plays a key role as the biocontrol agent against *L. theobromae* on macadamia, avocado, mango, and newly on wheat [24,25,26,27]. Thus, *Trichoderma* spp. possess the potential traits for preventing the growth of pathogens. Based on the possibility of *L. theobromae* species on pomelo in Ben Tre province, Vietnam, and the potential of *Trichoderma* species as an antagonist, the present study was conducted to (i) isolate pathogenic fungi causing pomelo stem-end rot disease (PSERD) and (ii) assess the antagonism of *Trichoderma* spp. against *Lasiodiplodia* spp. under in vitro conditions.

## 2. Materials and Methods

Time: The experiment was performed from January 2023 to August 2023.

Location: The experiment was conducted in the Microbiology Laboratory (10°01′45.5″ N 105°45′59.7′ E), Faculty of Crop Science, College of Agriculture, Can Tho University, Can Tho City, Vietnam.

### 2.1. Trichoderma spp. Source

Fifty *Trichoderma* spp. fungal strains labeled from T-SP01 to T-SP50 (our preliminary work) were isolated from the pomelo orchard’s soils in Ben Tre province (Table S1). They were maintained on the *Trichoderma* Selective Medium (TSM) containing 0.2 g MgSO_4_·7H_2_O, 1.18 g KH_2_PO_4_, 0.15 g KC1, 1.0 g NH_4_NO_3_, 0.5 g glucose, and 20 g agar, and distilled water for 1.0 L of total volume [28].

### 2.2. Isolation of Fungal Pathogens

Eleven pomelo fruits rotted with the symptoms of PSERD were collected from Chau Thanh and Binh Dai districts, Ben Tre province. These fruits were stored in cool conditions and transported to the Mycology Laboratory. In the laboratory, the fruits were surface-sterilized by 70% alcohol following the methods of Burgess et al. [29] and Shivas and Beasley [30]. The rotted tissues of the peel were cut into a square shape (2 × 2 mm), placed on the PDA medium, and then incubated at room temperature at roughly 28–30 °C under dark conditions. When the mycelia grew from the rotted tissue, they were immediately transferred into the new PDA medium. This step was conducted at least three times until the fungal isolates were pure. The mycelia of the fungal isolates were checked for purity using the CX23 microscope with 40x magnification (Olympus Corporation, Tokyo, Japan). Finally, the fungal isolates were utilized as material for Koch’s postulates test.

### 2.3. Evaluation of the Growth Rate of Fungal Pathogens and the Pathogenicity Test

When the fungal strains were 3 days old on the PDA medium, they were used to check for mycelial growth. From the grid of the mycelial growth, an agar plug (with a diameter of 6 mm) was cut and transferred into the new PDA plate and placed on the center of the plate, three replications for each fungal strain. The samples were incubated in the same conditions described above. The length of mycelial growth was observed at 24, 48, and 72 h after inoculation (HAI). Diameters of mycelia were measured twice on each plate. Fast-growing fungal strains were selected and re-inoculated into the healthy pomelo fruits following Koch’s postulates. Briefly, the healthy fruits were rinsed thoroughly with distilled water, put into the solution of 70% alcohol for 30 s, and rinsed again with sterile distilled water. The fruits were dried using sterile tissue paper. The fungal strains were grown on the PDA medium for three days, collected on the mycelium mat, and ground in the PDB medium using a hand mixer. This step was performed under laminar flow. The density of the fungal strain was adjusted to about 10^6^ CFU/mL. The pathogenicity test was conducted by injecting one milliliter fungal suspension (10^6^ CFU/mL) on an artificial wound on the peel of the sterilized fruit at three positions from the top, middle, and bottom of the fruit, with three replications of each position with a total of 9 injections for each pomelo. The negative control was also conducted by injecting 100 µL of sterile water. The pomelos were kept at room temperature in the dark and at 93% relative humidity for 15 days. The peels showing a rotted symptom were carefully photographed and used for fungal reisolation. With the confirmation of the Koch’s postulates test, the fast-growing fungal strains that caused the largest lesions on the fruit were utilized for the pathogenicity test.

### 2.4. Evaluation of Antagonistic Activity of Trichoderma spp. against Lasiodiplodia spp. In Vitro Test

Based on the results of the Koch’s postulates test, two fungal strains that caused severely rotted fruit were used as the two fungal pathogens. Fifty *Trichoderma* strains were provided from the Mycology Laboratory of the Faculty of Crop Science, College of Agriculture, Can Tho University, Vietnam. The dual-culture experiment was set up as a completely randomized design (CRD) with 50 treatments and 3 replications for each fungal pathogenic strain. Each *Trichoderma* strain was grown in dual culture with either the first fungal pathogen or the second pathogen. Each fungal pathogen and the *Trichoderma* strain were inoculated 6 cm apart on the same plate (9 cm diameter) and incubated at 27 °C under dark conditions. Radii of the colony of each pathogen approaching and not approaching the colony of the *Trichoderma* strain were measured twice at 42 and 72 h after inoculation (HAI). The antagonistic efficiency (AE) of the *Trichoderma* strains was evaluated at 48 and 72 HAI, as follows:

$$AE=\frac{C-T}{C}\times 100\%$$

where

AE is the antagonistic efficiency;
*C* is the radius of the pathogenic colony on PDA;
*T* is the radius of the pathogenic colony on PDA with *Trichoderma* spp.

The evaluation was performed according to the method of Kakraliya et al. [31]: AE ≥ 60% is highly antagonistic, 40% ≤ AE ≤ 59% is moderately antagonistic, 20% ≤ AE ≤ 39% is weakly antagonistic, and AE ≤ 19% is non-antagonistic.

### 2.5. Identification of the Pathogens and Trichoderma spp.

Selected strains of fungal pathogens and *Trichoderma* spp. were identified using the polymerase chain reaction (PCR) technique: DNA was extracted from hyphae of colonies after 7–10 days of culture on PDA. The hyphae were put in a 2.2 mL Eppendorf, shaken, and incubated at room temperature for 10 min. It was centrifuged at 13,000 rpm for 5 min. The extract was transferred into a new Eppendorf, and the precipitate was washed with 500 µL of ethanol 70%, centrifuged at 13,000 for 5 min, and vacuum dried. The DNA was dissolved in 100 µL TE 0.1X. The PCR was conducted with a pair of primers: ITS 1: 5′-TCCGTAGGTGAACCTGCGG-3′ and ITS 4: 5′-TCCTCCGCTTATTGATATGC-3′ [32]. The PCR reaction was performed in 50 µL volume via the following steps: denaturation (95 °C for 5 min), 30 cycles (95 °C for 90 s, annealing at 52 °C for 60 s, and elongation at 72 °C for 90 s), and termination at room temperature. PCR amplicons were purified and sequenced using an automatic sequencing machine. The result was compared with the GenBank database of the National Center for Biotechnology Information (NCBI) by using the Nucleotide Basic Local Alignment Search Tool (BLASTN) version 2.14.0 (National Library of Medicine, MD, USA). The multiple sequence alignments were performed using the ClustalW program in MEGA 6.0 software (Molecular Evolutionary Genetics Analysis, PA, USA). The neighbor-joining phylogenetic tree was built by using the MEGA 6.06 software, in which the evolutionary distance matrix was created by using the Jukes–Cantor model and topologies of the neighbor-joining trees were calculated by using the 1000-replication bootstrap [33].

### 2.6. Statistical Analysis

Collected numeric data were applied with one-way variance analysis (ANOVA) to determine the differences between fungal strains by Duncan’s test at 5% significance in the SPSS 13.0 software.

## 3. Results

### 3.1. Morphological Characteristics of Fungal Pathogen Causing PSERD

**Figure 1:**
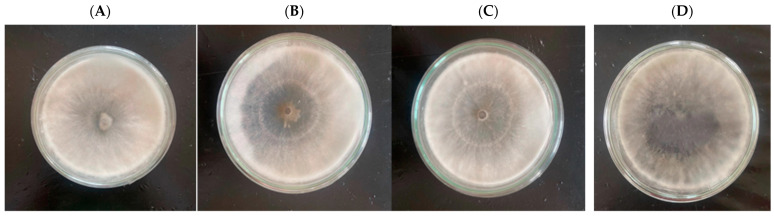
Morphological features of fungi causing pomelo stem-end rot disease on PDA. Note: (**A**) S-P03, (**B**) S-P04, (**C**) S-P06, and (**D**) S-P07.

Eleven fungal pathogen strains of PSERD were isolated on PDA medium from 11 fruit samples with the disease’s symptoms from pomelo farms in Ben Tre province (Table S1). All isolated fungal strains were fast-growing and covered a petri dish after 72 HAI. Their colonies were all circular. The mycelium mats were initially white and then turned dark gray or light gray at 120 HAI (Figure 1). The mycelia grew fast and followed uneven circular patterns at 7 days after inoculation (DAI). All strains had thickly growing hyphae, with concentric circles, and being water-proof. At 7 DAI, the mycelia were white and then turned blackish gray or black and covered all of the dish’s surface. The hyphae structure was diverse and porous and spread evenly. Some strains appeared with fungal blocks (Table 1).

### 3.2. Growth of Fungi Causing PSERD

At 24 HAI, the mycelia parameters differed at 5% significance. Therein, the S-P07 strain had the fastest growth diameter with 4.67 cm, while the others were from 1.07 to 4.40 cm. On the other hand, the slowest growth diameter belonged to the S-P09 strain. At 48 HAI, the S-P07 maintained the fastest growth (7.10 cm), while the S-P09 strain still had the lowest growth diameter (2.63 cm). The others grew from 4.70 to 6.37 cm. At 72 HAI, the S-P06 (8.67 cm) and S-P07 (8.77 cm) had the widest growth diameters. On the other hand, the S-P09 strain (5.33 cm) had the lowest one. Moreover, the other growth diameters were 6.10–8.67 cm (Table 2). Based on the mycelial growth, the two fungal strains S-P06 and S-P07 were selected for the antagonistic test under in vitro conditions.

### 3.3. Evaluation of the Infection by the Selected Fungi Causing PSERD

Pomelo fruits inoculated with the two selected pathogenic fungi all appeared with stem-end rot at 6–7 DAI, along with the diameter of the infection spots ranging from 1.05 to 1.25 cm. The whole fruit rot appeared at 12–15 DAI (Table 3).

### 3.4. Identification of Selected Pathogens of PSERD

**Figure 2:**
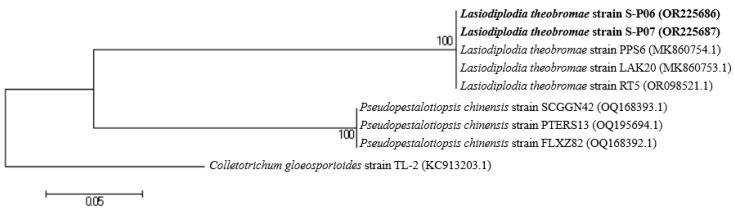
Neighbor-joining tree showing the phylogenetic relationships of S-P06 and S-P07 strains aligned with reference strains from the domain fungi based on their ITS sequences. The bold data are the name of the S-P06 and S-P07 strains along with their accession numbers. Bootstrap values are shown for nodes in a bootstrap analysis of 1000 replications. The scale bar indicates an estimated difference of 5% in the sequences.

The ITS gene sequencing indicated that the S-P06 and S-P07 strains were identified as *Lasiodiplodia theobromae* with accession numbers of OR225686 and OR225687, respectively, with 100% similarity (Figure 2). *Pseudopestalotiopsis chinensis* was utilized for comparison with *L. theobromae*, and the *Colletotrichum gloeosporioides* TL-2 strain was used as an outgroup (Figure 2).

### 3.5. Antagonistic Activity of Trichoderma Strains against Lasiodiplodia sp.

#### 3.5.1. Antagonistic Activity of *Trichoderma* sp. against *L. theobromae* S-P06

**Figure 3:**
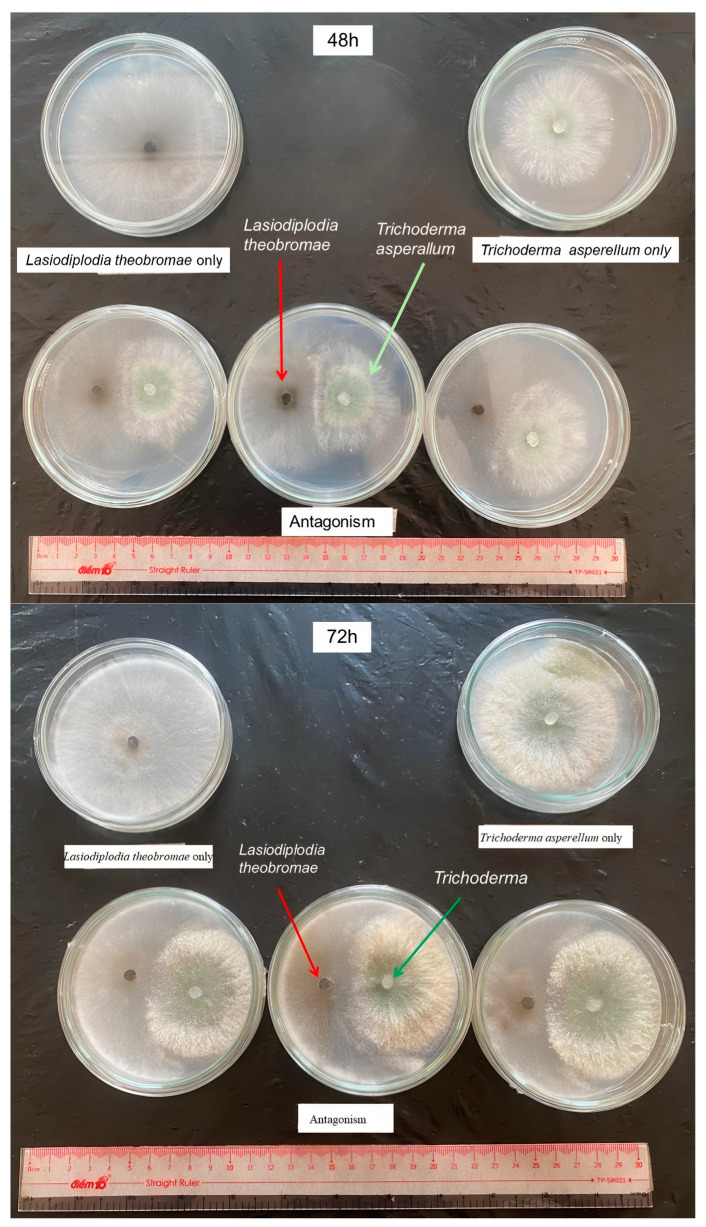
Antagonistic effect of *Trichoderma* sp. against *L. theobromae* S-P06 at 48 and 72 h after inoculation.

The suppression of pathogens on the PDA medium is illustrated in Figure 3. At 48 HAI, the AEs of the T-SP19 and T-SP32 strains were greater than the others (50.8% and 48.7%, respectively). At 72 HAI, the AEs of the *Trichoderma* strains against the *L. Theobromae* S-P06 fluctuated from moderate to high values, and the greatest values belonged to the T-SP19 and T-SP32 strains (86.2% and 85.4%, respectively). Moreover, the others had moderate control from 47.9% to 59.4% (Table 4).

#### 3.5.2. Antagonistic Capacity of Trichoderma sp. against *L. theobromae* S-P07 

**Figure 4:**
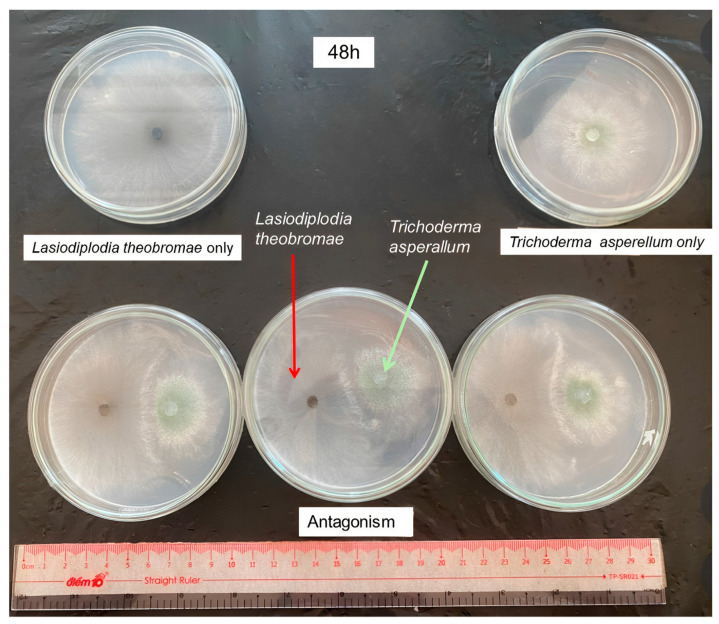

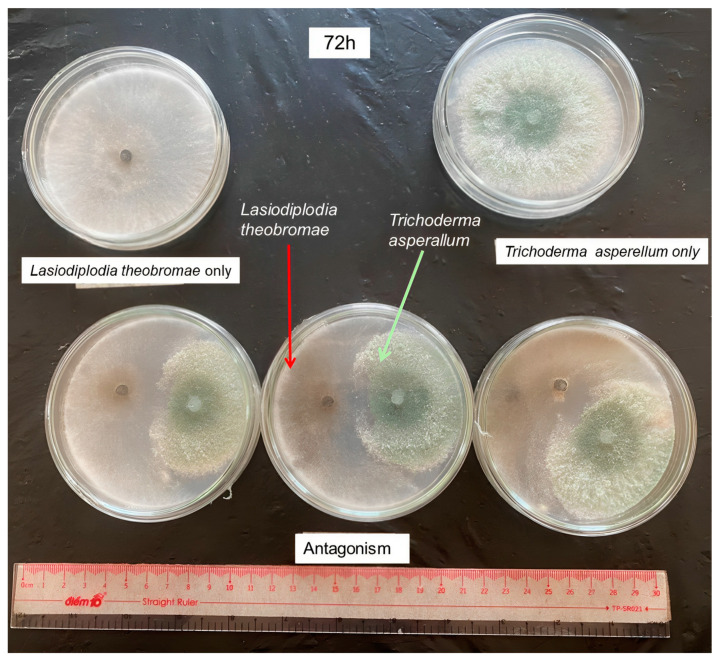
Antagonism of *Trichoderma* sp. against *L. theobromae* S-P07 on PDA at 48 and 72 h after inoculation.

At 48 HAI, *Trichoderma* spp. strains with moderate AEs were T-SP19 and T-SP32 (49.8% and 47.6%, respectively), whose results were greater than the others at 5% significance. Moreover, some weakly antagonistic strains included T-SP22, T-SP25, T-SP43, T-SP10, and T-SP39. At 72 HAI, AEs of *Trichoderma* sp. strains had moderate to high antagonism, especially T-SP19 and T-SP32 strains, with AEs at 85.4% and 84.7%, respectively. Furthermore, other strains’ AEs varied from 48.6% to 62.4% (Table 5). Figure 4 illustrates the antagonism on the PDA medium.

### 3.6. Identification of Trichoderma sp. Strains That highly Antagonized the L. theobromae S-P06 and L. theobromae S-P07 Strains

**Figure 5:**
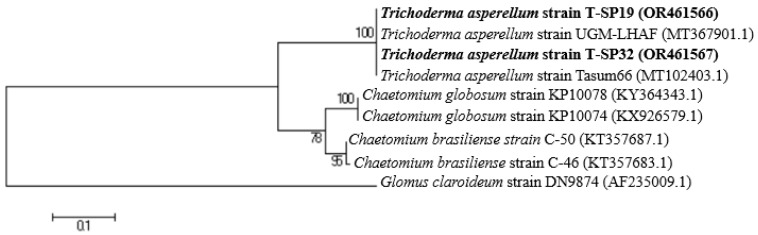
Neighbor-joining phylogenetic trees based on ITS sequences of three selected *Trichoderma* sp. strains in comparison with the closely related strains in the GenBank database. The bold data are the name of the T-SP19 and T-SP32 strains along with their accession numbers. The percentage levels of bootstrap analysis of 1000 replications are indicated at each node. Bar, 0.1 substitutions per nucleotide position. *Glomus claroideum* strain DN9874 was used as the outgroup strain. Access numbers of GenBank sequences are indicated in brackets.

The identification resulted in the two strongly antagonistic *Trichoderma* T-SP19 and T-SP32 strains against the *L. theobromae* S-P06 and S-P07 pathogens as *Trichoderma asperellum* T-SP19 and T-SP32, with 100% similarity, and their accession numbers as OR461566 and OR461567, respectively. The *Chaetomium* sp. genus was utilized as another fungal group, while the *Glomus claroideum* DN9874 strain was utilized as an outgroup (Figure 5).

## 4. Discussion

The PSERD-causing S-P06 and SP-07 strains was identified as *Lasiodiplodia theobromae* (Figure 1). The morphology of *L. theobromae* after isolation is described in Table 1. According to Gnanesh et al. [34], the morphology of *L. theobromae* causing root rot on strawberry consists of the color changing from white to gray and mycelia covering the dish surface after 4–5 DAI. After 14–15 DAI, the hyphae turned black, and the mycelia were blackish-gray, leaking some liquid which dried after a few days. A study of the morphology of *L. theobromae* infecting pomelo has shown that, at 4–7 DAI, white hyphae covered the dish surface. The mycelia of *L. theobromae* was initially white, then grew, turned whitish-green and black, and thickened on the dish surface at 21 DAI. Under a 40X microscope, the hyphae were transparent, then turned brown, and contained chlamydospore. Pycniospores developed within the pynidia. The pycniospores consisted of two layers and were granular. Pycniospores after maturity were slightly brown and had an intermediate wall separating the spore into two cells [35]. In Indonesia, species of *L. theobromae* have also been recently isolated from pomelo in different regions and shown identical morphological characteristics [35]. *L. theobromae* species are not only able to cause stem-end rot disease but also found to lead to leaf blight disease on rubber trees in Thailand. They are also found with similar morphology to that described above [36]. It can be stated that the morphology of *L. theobromae* species is identical.

At 72 HAI, the diameter of the mycelia of the *L. theobromae* was roughly 5.33–8.77 cm (Table 2). Briste et al. [37] have also isolated 2 *L. theobromae* strains causing stem-end rot disease. *L. theobromae* strains grew fast on PDA, with the mycelia diameter after 3 days of incubation being 86.8 mm for BU-DLa 01 and 79.6 mm for BU-DLa 02. Similarly, with *L. theobromae* as a pathogen on rain trees (*Samanea saman* (Jacq.) Merr.), hyphae reached their maximum growth on PDA at 72.11 mm and had high spore-producing efficiency, while under yeast extract agar (YEA) medium, the growth of hyphae decreased (59.19 mm) [38].

The two antagonists were identified was *Trichoderma asperellum* by ITS regions. This is in accordance with the study by Wu et al. [39], where a species of *T. asperellum* was simply identified by ITS. Moreover, in our study, the morphology of the *T. asperellum* was also observed and was identical to the morphology shown in the study by Samuels et al. [40], where distinguished morphologies of the three closely related *Trichoderma* species were illustrated as *T. asperellum*, *T. asperelloides*, and *T. yunnanense*. However, the use of ITS should be applied for identifying *Trichoderma* at genus level rather than species level [41]. The use of other regions (e.g., *tef*1) *s*hould be applied to distinguish *Trichoderma* species in several studies, where the sample size is big and ITS fails to show unambiguous identification [42], while our study worked on a much smaller sample size. Nevertheless, the lack of using other regions, such as *tef*1 and *rpb*2, is an unfortunate gap in our study, which should be filled in the following studies by our research group.

At 72 HAI, the *T. asperellum* T-SP19 and T-SP32 strains had the greatest AE against *L. theobromae* S-P06 at 86.2% and 85.4%, respectively (Table 4). In addition, the two T-SP19 and T-SP32 strains also had high AE against *L. theobromae* S-P07, with 85.4% and 84.7%, respectively (Table 5). Similarly, the inhibition of *T. hamatum* against *L. theobromae* harming macadamia nut was recorded up to 56.3% [24]. Furthermore, inoculating the suspension of *T. hamatum* spores on the wounds of macadamia trees resulted in lower disease severity index from 85.1% to 37.7% [24]. In the same line, the *Trichoderma harzianum* strain CE92 has been found to slow down the growth of *L. theobromae* species [43]. The *Trichoderma* sp. strains isolated from the Cerrado–Caatinga ecological forest suppressed the growth of *L. theobromae* hyphae from 30 to 78% [44]. It has been shown that the *T. longibrachiatum* and *T. asperellum* strains after 6 days of culture on PDA medium inhibited 86%–88% of the growth of *L. theobromae* causing stem-end rot disease on eucalyptus trees in Ethiopia. The *T. atroviride* strain had the lowest growth inhibition rate, at 58.5% [45]. For the stem-end rot disease caused by *L. theobromae* species, *Trichoderma* spp. (*T. atroviride, T. virens, T. asperellum,* and *T. harzianum*) have been applied to control the disease in avocado fruits [26]. Noticeably, on papaya, indigenous *Trichoderma* spp. are utilized for controlling the *L. theobromae* stem-end rot disease well [46]. In the current study, the two *Trichoderma* sp. T-SP19 and T-SP32 strains were isolated from soils for pomelo and used to antagonize the *L. theobromae* infecting the pomelo fruits on those soils. Furthermore, *Trichoderma* sp. can also inhibit the growth of *L. theobromae* in other circumstances. For instance, the antagonism of *T. asperelloides*, *T. asperellum*, and *T. koningiopsis* against *L. theobromae* damaging the punching marks on grape trees has been also recorded by Marraschi et al. [47]. *T. asperelloides* is highly considered in controlling *L. theobromae* pathogens, equivalent to the efficiency of fungicides such as fuazinam and tebuconazole at 10 and 20 DAI. The antagonistic mechanisms of *Trichoderma* spp. comprise producing secondary metabolites, parasitizing, and inhibiting the pathogens. Hyphae of *Trichoderma* spp. can produce chitinase, glucanase, and protease, which disfacilitate fungal hosts. *T. atroviride* produces Gliotoxin, suppressing the growth of pathogenic hyphae [48]. Similarly, according to Bedine et al. [46], strains of *Trichoderma* spp. produce volatile and non-volatile secondary metabolites to inhibit the hyphae growth of *L. theobromae* causing root and treetop rot on papaya tress at 68.2% (*T. harzianum* BRS-7), 68.2% (*T. koningiopsis* BRS-9), 53.5% (*T. harzianum BRS-8*), and 53.4% (*T. asperellum* BRS-1). Additionally, strains of *T. asperellum* BRS-1 and *T. harzianum* BRS-7 can antagonize *L. theobromae* under in vitro conditions in 9 days, with a growth rate drop of 75.4 and 64.1%, respectively. Ultimately, *Trichoderma* species are such powerful tools to limit the spread of *L. theobromae*-related diseases among tropical and subtropical regions. Thereby, the two promising *T. asperellum* T-SP19 and T-SP32 strains should be further tested to control PSERD, and possibly other diseases caused by *L. theobromae in vivo*, and compared with some commercialized chemical fungicides or bio-fungicides.

## 5. Conclusions

Two out of eleven PSERD-causing strains were selected for strongly exhibiting the disease’s symptoms and were identified as *Lasiodiplodia theobromae* S-P06 and S-P07. Two *Trichoderma asperellum* strains were identified, T-SP19 and T-SP32, and selected from fifty *Trichoderma* strains for greatly antagonizing the *L. theobromae* S-P06 and S-P07 strains, with AEs above 84%. These two new antagonists should be further studied under in vivo conditions to control the PSERD as compared to a commercial local bio-fungicide product containing *Trichoderma* species (DHCT-Tricho, in particular).

## Figures and Tables

**Table 1:** Morphology of fungi causing pomelo stem-end rot disease on PDA medium at 7 days after inoculation.

Strains	Mycelia Color
S-P01	White hyphae turned dark gray
S-P02	White hyphae turned dark gray
S-P03	White hyphae turned totally dark gray on the dish
S-P04	White hyphae turned grayish-black and were lighter from the border to the center of the dish, with many fungal blocks
S-P05	White hyphae turned unevenly grayish-black
S-P06	White hyphae turned grayish-black and were lighter from the border to the center of the dish, with many fungal blocks
S-P07	White hyphae turned grayish-black alternatively from the border to the center of the dish, with many fungal blocks
S-P08	White hyphae turned unevenly grayish-black
S-P09	White hyphae turned dark gray
S-P10	White hyphae turned totally dark gray on the dish
S-P11	White hyphae turned dark gray

**Table 2:** Growth of fungi causing pomelo stem-end rot disease on PDA.

Strains	Mycelial Length of Fungal Pathogen Grown on the Medium after Transferring (cm)		
	24 h	48 h	72 h
S-P01	3.10 ^f^	4.57 ^f^	6.10 ^h^
S-P02	2.63 ^g^	5.27 ^e^	7.40 ^f^
S-P03	4.00 ^c^	5.60 ^d^	8.00 ^c^
S-P04	4.17 ^bc^	6.13 ^bc^	8.37 ^b^
S-P05	3.57 ^e^	5.60 ^d^	7.60 ^e^
S-P06	4.40 ^b^	6.37 ^b^	8.67 ^a^
S-P07	4.67 ^a^	7.10 ^a^	8.77 ^a^
S-P08	4.03 ^c^	6.13 ^bc^	7.80 ^d^
S-P09	1.07 ^h^	2.63 ^g^	5.33 ^i^
S-P10	3.73 ^de^	5.73 ^d^	7.20 ^g^
S-P11	3.97 ^cd^	6.03 ^c^	8.30 ^b^
Level of significance	*	*	*
CV (%)	4.10	2.47	1.80

Note: In the same column, numbers followed by the same letters are insignificantly different following Duncan’s test. (*) different at 5%. *n* = 3.

**Table 3:** Time of appearance of fruit rot sign, whole fruit rot, and diameter of fruit rot.

Fungal Pathogen	Time for the Symptom to Appear (DAI)	Time of Whole Fruit Rot (DAI)	Diameter of Fruit Rot (cm)
S-P06	7	15	1.25
S-P07	6	12	1.05
Control	No fruit rot	No fruit rot	No fruit rot

**Table 4:** Antagonistic efficiency of *Trichoderma* sp. against *L. theobromae* S-P06.

No.	Strains	Antagonistic Efficiency (%)		Diameter of Antagonism against *L. theobromae* S-P06 (cm)	
		48 h	72 h	48 h	72 h
1	T-SP01	22.5 ^bc^	52.2 ^f–k^	2.47 ^a^ ± 0.06	2.07 ^b–f^ ± 0.06
2	T-SP02	20.8 ^bc^	54.0 ^c–i^	2.53 ^a^ ± 0.0	2.00 ^c–g^ ± 0.15
3	T-SP03	22.9 ^bc^	52.3 ^f–k^	2.47 ^a^ ± 0.06	2.07 ^b–f^ ± 0.12
4	T-SP04	20.8 ^bc^	56.1 ^b–g^	2.53 ^a^ ± 0.06	1.90 ^f–j^ ± 0.12
5	T-SP05	21.9 ^bc^	53.2 ^d–j^	2.50 ^a^ ± 0.10	2.03 ^b–g^ ± 0.10
6	T-SP06	22.5 ^bc^	48.6 ^jk^	2.47 ^a^ ± 0.06	2.23 ^ab^ ± 0.12
7	T-SP07	20.4 ^bc^	50.2 ^ijk^	2.53 ^a^ ± 0.06	2.17 ^a–d^ ± 0.06
8	T-SP08	19.8 ^bc^	53.3 ^d–j^	2.57 ^a^ ± 0.06	2.03 ^b–g^ ± 0.12
9	T-SP09	22.9 ^bc^	56.3 ^b–f^	2.47 ^a^ ± 0.10	1.90 ^f–j^ ± 0.06
10	T-SP10	19.4 ^bc^	50.2 ^i–k^	2.57 ^a^ ± 0.06	2.17 ^a–d^ ± 0.15
11	T-SP11	22.1 ^bc^	55.7 ^b–g^	2.47 ^a^ ± 0.06	1.93 ^e–i^ ± 0.10
12	T-SP12	20.6 ^bc^	55.7 ^b–g^	2.50 ^a^ ± 0.06	1.93 ^e–i^ ± 0.15
13	T-SP13	21.0 ^bc^	52.9 ^e–j^	2.50 ^a^ ± 0.06	2.03 ^b–g^ ± 0.06
14	T-SP14	21.7 ^bc^	55.2 ^b–h^	2.47 ^a^ ± 0.10	1.93 ^e–i^ ± 0.10
15	T-SP15	20.0 ^bc^	56.0 ^b–g^	2.53 ^a^ ± 0.06	1.90 ^f–j^ ± 0.06
16	T-SP16	22.9 ^bc^	54.0 ^c–i^	2.47 ^a^ ± 0.06	2.00 ^c–g^ ± 0.06
17	T-SP17	22.5 ^bc^	57.8 ^b–d^	2.47 ^a^ ± 0.06	1.83 ^h–j^ ± 0.12
18	T-SP18	20.0 ^bc^	59.4 ^b^	2.53 ^a^ ± 0.06	1.77 ^i^ ± 0.10
19	T-SP19	50.8^a^	86.2 ^a^	1.57 ^b^ ± 0.10	0.60 ^j^ ± 0.06
20	T-SP20	21.3 ^bc^	52.1 ^f–k^	2.47 ^a^ ± 0.06	2.07 ^b–f^ ± 0.12
21	T-SP21	21.5 ^bc^	49.8 ^ijk^	2.50 ^a^ ± 0.06	2.17 ^a–d^ ± 0.12
22	T-SP22	18.1^c^	54.4 ^c–i^	2.57 ^a^ ± 0.15	1.97 ^d–h^ ± 0.12
23	T-SP23	21.9 ^bc^	52.1 ^f–k^	2.50 ^a^ ± 0.15	2.07 ^b–f^ ± 0.06
24	T-SP24	22.9 ^bc^	52.7 ^e–j^	2.47 ^a^ ± 0.06	2.03 ^b–g^ ± 0.06
25	T-SP25	19.0 ^bc^	50.4 ^h–k^	2.57 ^a^ ± 0.15	2.13 ^a–e^ ± 0.15
26	T-SP26	21.1 ^bc^	57.5 ^b–e^	2.50 ^a^ ± 0.06	1.83 ^h–j^ ± 0.06
27	T-SP27	21.4 ^bc^	51.3 ^g–k^	2.50 ^a^ ± 0.10	2.10 ^a–f^ ± 0.10
28	T-SP28	22.1 ^bc^	52.9 ^e–j^	2.47 ^a^ ± 0.06	2.03 ^b–g^ ± 0.10
29	T-SP29	20.4 ^bc^	49.0 ^jk^	2.53 ^a^ ± 0.06	2.20 ^a–c^ ± 0.12
30	T-SP30	21.1 ^bc^	52.1 ^f–k^	2.50 ^a^ ± 0.06	2.07 ^b–f^ ± 0.15
31	T-SP31	22.1 ^bc^	47.9 ^k^	2.47 ^a^ ± 0.06	2.27 ^a^ ± 0.12
32	T-SP32	48.7^a^	85.4 ^a^	1.63 ^b^ ± 0.10	0.63 ^j^ ± 0.15
33	T-SP33	22.5 ^bc^	49.6 ^i–k^	2.47 ^a^ ± 0.12	2.17 ^a–d^ ± 0.15
34	T-SP34	20.0 ^bc^	50.4 ^h–k^	2.53 ^a^ ± 0.15	2.13 ^a–e^ ± 0.12
35	T-SP35	22.1 ^bc^	54.3 ^c–i^	2.47 ^a^ ± 0.06	1.97 ^d–h^ ± 0.10
36	T-SP36	20.6 ^bc^	58.3 ^b c^	2.50 ^a^ ± 0.17	1.80 ^hi^ ± 0.15
37	T-SP37	22.1 ^bc^	52.9 ^e–j^	2.47 ^a^ ± 0.06	2.03 ^b–g^ ± 0.10
38	T-SP38	22.5 ^bc^	51.3 ^g–k^	2.47 ^a^ ± 0.15	2.10 ^a–f^ ± 0.15
39	T-SP39	19.4 ^bc^	52.1 ^f–k^	2.57 ^a^ ± 0.17	2.07 ^b–f^ ± 0.15
40	T-SP40	21.9 ^bc^	50.6 ^h–k^	2.50 ^a^ ± 0.12	2.13 ^a–e^ ± 0.12
41	T-SP41	24.0 ^bc^	52.9 ^e–j^	2.43 ^a^ ± 0.06	2.03 ^b–g^ ± 0.12
42	T-SP42	21.9 ^bc^	55.2 ^b–h^	2.50 ^a^ ± 0.12	1.93 ^e–i^ ± 0.12
43	T-SP43	19.0 ^bc^	54.4 ^c–i^	2.57 ^a^ ± 0.15	1.97 ^d–h^ ± 0.15
44	T-SP44	22.1 ^bc^	52.1 ^f–k^	2.47 ^a^ ± 0.12	2.07 ^b–f^ ± 0.10
45	T-SP45	20.8 ^bc^	52.1 ^f–k^	2.53 ^a^ ± 0.06	2.07 ^b–f^ ± 0.10
46	T-SP46	24.0 ^bc^	53.5 ^d–j^	2.43 ^a^ ± 0.06	2.00 ^c–g^ ± 0.06
47	T-SP47	22.9 ^bc^	52.7 ^e–j^	2.47 ^a^ ± 0.15	2.03 ^b–g^ ± 0.12
48	T-SP48	20.0 ^bc^	49.6 ^i–k^	2.53 ^a^ ± 0.12	2.17 ^a–d^ ± 0.15
49	T-SP49	22.1 ^bc^	52.7 ^e–j^	2.47 ^a^ ± 0.06	2.03 ^b–g^ ± 0.12
50	T-SP50	22.1 ^bc^	52.7 ^e–j^	2.47 ^a^ ± 0.12	2.03 ^b–g^ ± 0.12
F		*	*	*	*
CV (%)		11.3	4.43	2.8	5.01

Note: In the same column, numbers followed by the same letters are insignificantly different via Duncan’s test. (*) different at 5%. T: *Trichoderma*; S: stem-end; P: pomelo. *n* = 3.

**Table 5:** Antagonistic efficiency of *Trichoderma* sp. against *L. theobromae* S-P07.

No.	Strains	Antagonistic Efficiency (%)		Diameter of Antagonism against *L. theobromae* S-P07 (cm)	
		48 h	72 h	48 h	72 h
1	T-SP01	22.5 ^b–d^	50.4 ^k–n^	2.47 ^ab^ ± 0.10	2.17 ^a–c^ ± 0.10
2	T-SP02	20.8 ^cd^	54.8 ^d–j^	2.53 ^a^ ± 0.06	1.97 ^d–g^ ± 0.15
3	T-SP03	22.9 ^b–d^	52.3 ^f–n^	2.47 ^ab^ ± 0.10	2.07 ^a–e^ ± 0.12
4	T-SP04	27.0 ^b^	55.4 ^d–g^	2.33 ^b^ ± 0.06	1.93 ^e–g^ ± 0.12
5	T-SP05	21.9 ^b–d^	54.7 ^d–k^	2.50 ^a^ ± 0.10	1.97 ^d–g^ ± 0.10
6	T-SP06	22.5 ^b–d^	48.6 ^n^	2.47 ^ab^ ± 0.15	2.23 ^a^ ± 0.15
7	T-SP07	20.4 ^cd^	51.0 ^h–n^	2.53 ^a^ ± 0.10	2.13 ^a–d^ ± 0.10
8	T-SP08	19.8 ^cd^	54.8 ^e–i^	2.57 ^a^ ± 0.15	1.97 ^d–g^ ± 0.10
9	T-SP09	22.9 ^b–d^	54.8 ^d–j^	2.47 ^ab^ ± 0.10	1.97 ^d–g^ ± 0.15
10	T-SP10	19.4 ^cd^	51.7 ^f–n^	2.57 ^a^ ± 0.10	2.10 ^a–e^ ± 0.06
11	T-SP11	22.1 ^b–d^	54.2 ^e–k^	2.47 ^ab^ ± 0.10	2.00 ^c–f^ ± 0.06
12	T-SP12	20.6 ^cd^	55.7 ^c–f^	2.50 ^a^ ± 0.06	1.93 ^efg^ ± 0.06
13	T-SP13	21.0 ^cd^	52.1 ^f–n^	2.50 ^a^ ± 0.10	2.07 ^a–e^ ± 0.12
14	T-SP14	21.7 ^cd^	54.4 ^e–k^	2.47 ^ab^ ± 0.15	1.97 ^d–g^ ± 0.10
15	T-SP15	20.0 ^cd^	55.2 ^d–h^	2.5 ^a^ ± 0.10	1.93 ^e–g^ ± 0.12
16	T-SP16	22.9 ^b–d^	53.2 ^f–m^	2.47 ^ab^ ± 0.10	2.03 ^b–e^ ± 0.06
17	T-SP17	22.5 ^b–d^	58.6 ^cd^	2.47 ^ab^ ± 0.15	1.80 ^gh^ ± 0.06
18	T-SP18	20.0 ^cd^	62.4 ^b^	2.53 ^a^ ± 0.06	1.63 ^i^ ± 0.06
19	T-SP19	49.8 ^a^	85.4 ^a^	1.60 ^c^ ± 0.15	0.63 ^j^ ± 0.15
20	T-SP20	21.3 ^cd^	52.9 ^f–n^	2.47 ^ab^ ± 0.15	2.03 ^b–e^ ± 0.10
21	T-SP21	21.5 ^cd^	51.4 ^f–n^	2.50 ^a^ ± 0.10	2.10 ^a–e^ ± 0.06
22	T-SP22	18.1 ^d^	54.4 ^e-k^	2.57 ^a^ ± 0.06	1.97 ^d–g^ ± 0.10
23	T-SP23	21.9 ^b–d^	52.1 ^f–n^	2.50 ^a^ ± 0.06	2.07 ^a–e^ ± 0.10
24	T-SP24	22.9 ^b–d^	53.5 ^e-l^	2.47 ^ab^ ± 0.12	2.00 ^c–f^ ± 0.12
25	T-SP25	19.0 ^cd^	50.4 ^j-n^	2.57 ^a^ ± 0.06	2.13 ^a–d^ ± 0.12
26	T-SP26	21.1 ^cd^	57.5 ^c–e^	2.50 ^a^ ± 0.10	1.83 ^f–h^ ± 0.10
27	T-SP27	21.4 ^cd^	51.3 ^f–n^	2.50 ^a^ ± 0.10	2.10 ^a–e^ ± 0.15
28	T-SP28	22.1 ^b–d^	52.9 ^f–n^	2.47 ^ab^ ± 0.10	2.03 ^b–e^ ± 0.06
29	T-SP29	20.4 ^cd^	49.0 ^mn^	2.53 ^a^ ± 0.10	2.20 ^ab^ ± 0.12
30	T-SP30	21.1 ^cd^	52.9 ^f–n^	2.50 ^a^ ± 0.15	2.03 ^b–e^ ± 0.15
31	T-SP31	22.1 ^b–d^	51.3 ^f–n^	2.47 ^ab^ ± 0.06	2.10 ^a–e^ ± 0.15
32	T-SP32	47.6 ^a^	84.7 ^a^	1.67 ^c^ ± 0.10	0.67 ^j^ ± 0.15
33	T-SP33	22.5 ^b–d^	51.2 ^g–n^	2.47 ^ab^ ± 0.12	2.10 ^a–e^ ± 0.12
34	T-SP34	20.0 ^cd^	50.4 ^j-n^	2.53 ^a^ ± 0.06	2.13 ^a–d^ ± 0.06
35	T-SP35	22.1 ^b–d^	54.2 ^e-k^	2.47 ^ab^ ± 0.10	1.97 ^d–g^ ± 0.12
36	T-SP36	20.6 ^cd^	59.4 ^bc^	2.50 ^a^ ± 0.06	1.77 ^hi^ ± 0.12
37	T-SP37	22.1 ^b–d^	52.9 ^f–n^	2.47 ^ab^ ± 0.06	2.03 ^b–e^ ± 0.06
38	T-SP38	22.5 ^b–d^	51.3 ^f–n^	2.47 ^ab^ ± 0.06	2.10 ^a–e^ ± 0.12
39	T-SP39	19.4 ^cd^	52.1 ^f–n^	2.57 ^a^ ± 0.10	2.07 ^a–e^ ± 0.12
40	T-SP40	21.9 ^b–d^	50.6 ^i–n^	2.50 ^a^ ± 0.06	2.13 ^a–d^ ± 0.06
41	T-SP41	24.0 ^bc^	52.9 ^f–n^	2.43 ^ab^ ± 0.10	2.03 ^b–e^ ± 0.10
42	T-SP42	21.9 ^b–d^	55.2 ^d–h^	2.50 ^a^ ± 0.10	1.93 ^e–g^ ± 0.12
43	T-SP43	19.0 ^cd^	52.5 ^f–n^	2.57 ^a^ ± 0.10	2.07 ^a–e^ ± 0.15
44	T-SP44	22.1 ^b–d^	52.9 ^f–n^	2.47 ^ab^ ± 0.15	2.03 ^b–e^ ± 0.10
45	T-SP45	20.8 ^cd^	52.1 ^f–n^	2.53 ^a^ ± 0.06	2.07 ^a–e^ ± 0.02
46	T-SP46	24.0 ^bc^	53.5 ^e–l^	2.43 ^ab^ ± 0.10	2.00 ^c–f^ ± 0.12
47	T-SP47	22.9 ^b–d^	52.7 ^f–n^	2.47 ^ab^ ± 0.10	2.03 ^b–e^ ± 0.06
48	T-SP48	20.0 ^cd^	49.6 ^l–n^	2.53 ^a^ ± 0.06	2.17 ^a–c^ ± 0.10
49	T-SP49	22.1 ^b–d^	52.7 ^f–n^	2.47 ^ab^ ± 0.15	2.03 ^b–e^ ± 0.10
50	T-SP50	22.1 ^b–d^	52.7 ^f–n^	2.47 ^ab^ ± 0.10	2.03 ^b–e^ ± 0.15
F		*	*	*	*
CV (%)		11.4	3.90	2.80	4.54

Note: In the same column, numbers followed by the same letters are insignificantly different via Duncan’s test. (*) different at 5%. T: *Trichoderma*; S: stem-end; P: pomelo. *n* = 3.

## Data Availability

The data presented in this study are available on request from the corresponding author.

## Supplementary Materials

The following supporting information can be downloaded at https://www.mdpi.com/article/10.3390/jof9100981/s1: Figure S1: Pomelo fruit with symptoms of stem-end rot disease after 5 days’ incubation. Table S1: Coordinates of isolating the causing factors of pomelo stem-end rot disease and *Trichoderma* antagonists.
