# Supplementary material for: Using Trichoderma asperellum to Antagonize Lasiodiplodia theobromae Causing Stem-End Rot Disease on Pomelo (Citrus maxima)
Source: J Fungi (Basel). 2023 Sep 29;9(10):981. doi: 10.3390/jof9100981 (PMC10607552; doi:10.3390/jof9100981)
Supplement: Supplementary file 1 [file jof-09-00981-s001.zip › jof-2594372-supplementary.pdf]

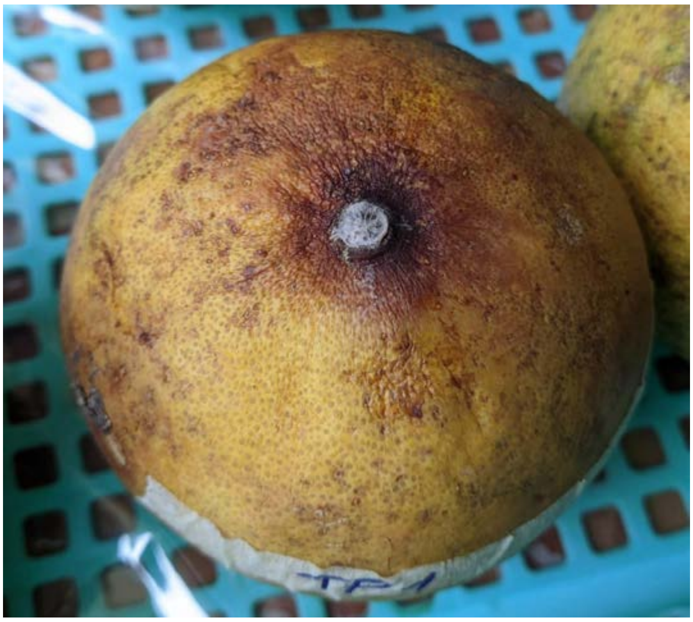

Figure S1: Pomelo fruit with symptoms of stem-end rot disease after 5 days incubation.

Table S1: Coordinates of isolating the causing factors of pomelo stem end rot disease and *Trichoderma* antagonists.

No.	Pathogenic strains	*Trichoderma* strain	Coordinate
1		T-SP01<br>T-SP02<br>T-SP03<br>T-SP04<br>T-SP21<br>T-SP22	10°10'25.8"N 106°29'05.7"E<br>10.173833, 106.484908<br>Binh Hoa commune, Giong Trom district, Ben Tre province
2		T-SP05<br>T-SP06	10°11'04.9"N 106°22'43.9"E<br>10.184680, 106.378864<br>Son Phu commune, Giong Trom district, Ben Tre province
3		T-SP07<br>T-SP08	10°11'15.2"N 106°29'02.4"E<br>10.187558, 106.483993<br>Binh Hoa commune, Giong Trom district, Ben Tre province
4		T-SP09<br>T-SP10	10°14'51.3"N 106°26'54.5"E<br>10.247594, 106.448467<br>Phong Nam commune, Giong Trom district, Ben Tre province
5		T-SP11<br>T-SP12	10°10'53.0"N 106°23'04.8"E<br>10.181387, 106.384671<br>Son Phu commune, Giong Trom district, Ben Tre province
6		T-SP13<br>T-SP14	10°11'17.6"N 106°29'30.4"E<br>10.188221, 106.491771<br>Binh Hoa commune, Giong Trom district, Ben Tre province
7		T-SP15<br>T-SP16	10°10'54.4"N 106°29'01.7"E<br>10.181784, 106.483806<br>Binh Hoa commune, Giong Trom district, Ben Tre province
8		T-SP17<br>T-SP18	10°10'35.6"N 106°28'48.5"E<br>10.176558, 106.480144<br>Binh Hoa commune, Giong Trom district, Ben Tre province
9		T-SP19<br>T-SP20	10°10'38.9"N 106°28'49.0"E<br>10.177460, 106.480267<br>Binh Hoa commune, Giong Trom district, Ben Tre province
10		T-SP23<br>T-SP24	10°17'16.9"N 106°19'46.3"E<br>10.288025, 106.329513<br>Tam Phuoc commune, Chau Thanh district, Ben Tre province
11		T-SP25	10°16'54.7"N 106°19'50.0"E

		T-SP26	10.281861, 106.330541<br>Tam Phuoc commune, Chau Thanh district, Ben Tre province
12		T-SP27<br>T-SP28	10°17'33.9"N 106°19'46.7"E<br>10.292759, 106.329640<br>Tam Phuoc commune, Chau Thanh district, Ben Tre province
13		T-SP29<br>T-SP30	10°17'41.0"N 106°19'32.6"E<br>10.294708, 106.325734<br>Tam Phuoc commune, Chau Thanh district, Ben Tre province
14	S-P08	T-SP31<br>T-SP32	10°17'21.4"N 106°13'56.0"E<br>10.289266, 106.232224<br>Quoi Thanh commune, Chau Thanh district, Ben Tre province
15		T-SP33<br>T-SP34	10°17'37.1"N 106°14'12.4"E<br>10.293649, 106.236786<br>Quoi Thanh commune, Chau Thanh district, Ben Tre province
16		T-SP35<br>T-SP36	10°17'36.1"N 106°14'21.6"E<br>10.293350, 106.239330<br>Quoi Thanh commune, Chau Thanh district, Ben Tre province
17	S-P09	T-SP37<br>T-SP38	10°17'21.8"N 106°14'14.1"E<br>10.289384, 106.237235<br>Quoi Thanh commune, Chau Thanh district, Ben Tre province
18		T-SP39<br>T-SP40	10°15'52.9"N 106°13'04.0"E<br>10.264684, 106.217763<br>Tien Long commune, Chau Thanh district, Ben Tre province
19	S-P02	T-SP41<br>T-SP42	10°15'32.3"N 106°12'38.5"E<br>10.258964, 106.210691<br>Tien Long commune, Chau Thanh district, Ben Tre province
20	S-P01	T-SP43<br>T-SP44	10°15'50.3"N 106°13'51.7"E<br>10.263973, 106.231028<br>Tien Long commune, Chau Thanh district, Ben Tre province
21	S-P05	T-SP45<br>T-SP46	10°15'37.6"N 106°13'54.6"E<br>10.260435, 106.231824<br>Tien Thuy commune, Chau Thanh district, Ben Tre province
22	S-P04<br>S-P06<br>S-P07	T-SP47<br>T-SP48	10°15'34.0"N 106°13'52.5"E<br>10.259446, 106.231261<br>Tien Thuy commune, Chau Thanh district, Ben Tre province
23	S-P03	T-SP49<br>T-SP50	10°15'39.2"N 106°13'55.8"E<br>10.260887, 106.232177<br>Tien Thuy commune, Chau Thanh district, Ben Tre province
			10°15'43.5"N 106°27'45.5"E

24	S-P10	10.262069, 106.462636<br>Long Hoa commune, Binh Dai district,<br>Ben Tre province
25	S-P11	10°15'54.2"N 106°27'45.1"E<br>10.265066, 106.462539<br>Long Hoa commune, Binh Dai district,<br>Ben Tre province
